# Thioflavin-T: application as a neuronal body and nucleolar stain and the blue light photo enhancement effect

**DOI:** 10.1038/s41598-024-74359-8

**Published:** 2024-10-22

**Authors:** Jin-Hong Min, Heela Sarlus, Sho Oasa, Robert A. Harris

**Affiliations:** Department of Clinical Neuroscience, Karolinska Institutet, Center for Molecular Medicine, Karolinska University Hospital, 171 76 Stockholm, Sweden

**Keywords:** Thioflavin-T, Nissl, Neuron, Photo Enhancement, Brain, Amyloid Beta, Alzheimer’s disease, Fluorescence imaging, Cellular neuroscience, Confocal microscopy, Wide-field fluorescence microscopy

## Abstract

Thioflavin-T (THT) is a common and indispensable tool for the study of amyloid pathologies and protein aggregation, both in vitro and for histological samples. In this study we expand the use of THT beyond its canonical usage for staining amyloid plaques and demonstrate its novel use as an easy and rapid stain comparable to fluorescent Nissl staining, allowing for clear discernment of neuronal cell bodies and also nucleoli in fixed tissue and live cells. We believe that this is of value for any lab that studies central nervous system (CNS) tissues. Furthermore, we show that THT could potentially be used as a an alternative to the use of fluorescent reporters or other more costly RNA binding compounds in the study of nucleolar dynamics owing to its ability to clearly stain nucleoli in live cells. We also discovered the previously unreported effect of blue light exposure on the photo enhancement of THT excited by a 488 nm laser in stained tissue sample and how to avoid complications arising from this effect. Finally, we provide a simple protocol that can be easily adjusted either for using THT as a neuronal cell body and nucleoli stain, compatible with antibody based staining methods tested up to 4 fluorophores, or alternatively by using an additional washing step the protocol may be used for amyloid plaque detection in fixed brain tissue.

## Introduction

Thioflavin-T (THT) is a benzothiazole dye that is widely used in the study of amyloid fibril formation and is considered a ‘gold standard’ in this respect both in vitro and *in vivo*^1,2^*.* THT fluorescence occurs as a result of its binding to grooves within β-sheets which are common to amyloid fibrils. Upon binding and excitation at 450 nm it fluoresces brightly at around 482 nm wavelength due the immobilization of its rotary structure^3–5^. This property has led to its widespread applications as a fluorescent marker for the quantification of many different types of amyloid fibrils. Applications include in vitro fibrillation assays and the histological staining of amyloid structures in neurodegenerative diseases such as Alzheimer’s disease (AD), Prion disease (PrD), Parkinson’s disease (PD) and Amyotrophic Lateral Sclerosis (ALS), each of which has protein aggregation as a core aspect of disease pathology^6–8^.

Surprisingly, with a few exceptions, the use of THT is largely confined to in vitro fibrillation assays using purified peptides, and has not seen wide adoption for histology despite advantages over other traditionally used dyes such as Congo red^3,7,9^. In this regard, thioflavin S (THS) is more commonly employed for detecting amyloid pathologies in histological samples^10,11^. Although it is related to THT, THS in fact a heterogeneous mixture of benzothiazole compounds that mainly comprises of a dibenzothiazole molecule^12^, and unlike THT does not undergo a spectral shift when binding to amyloids^1^, which has generally excluded its use in in vitro fibrillation assays. The reason for THT being less used for histological amyloid detection may in part be due to THS being well established in this capacity, but also for the ability of THT to distinctly stain non-amyloid structures which we elucidate here.

This non-amyloid binding in a complex substrate such as fixed tissue, may be in part due to THT not being fully specific to β-sheets, as it can also bind to DNA base pairs, hyaline cartilage, human serum albumin and α-helices present in acetylcholinesterase^13–16^. This complicates its use as a fluorescent stain in tissue samples, as one must be careful to draw conclusions about whether or not a positive signal is derived from β-sheet-rich aggregates or else due to non-specific tissue binding. However, this has also enabled the application of THT beyond this canonical usage in amyloid detection. One example is the alternative use of THT is in the detection of endoplasmic reticulum (ER) stress, both in vitro and on fixed liver samples, or as an indicator of mitochondrial membrane potential and nucleoli^17–20^.

It is interesting to note that both the ER and nucleoli act as a sites of protein quality control that handle misfolded proteins. In the case of the ER it is able to retain partially folded and misfolded proteins, preventing their export which in some cases may even aggregate in the ER. However this is often connected to a diseased state^21^, and similarly under stress the nucleolus accumulates misfolded nuclear proteins for storage that limits the irreversible aggregation of these proteins^22,23^. These properties could allow susceptibility to THT staining, especially under conditions of cell stress, as demonstrated by protein aggregate detection during ER stress^18^, but does not explain the widespread staining evident that we will demonstrate later in young and healthy mice. A far more likely reason to explain this binding is that THT can bind mRNA and has been used to study mRNA metabolism and this binding mRNA rich structures is reported^23^. Furthermore THT has also been reported to have binding affinity to basophilic elements^14^. This feature of THT to stain both the nucleolus and ER is strikingly similar to Nissl staining. The principle of Nissl staining requires the use of basic dyes such as toluidine blue or cresyl violet to bind to the Nissl substance RNA content of cells^24^ which is evident in the ER of neurons that are rich in ribosomes with a high protein synthesis rate, thereby selectively labelling these neurons^25^. The Nissl staining technique is one of the earliest stains for neuronal cell bodies and has been used for over a century for selective staining in brain samples^26,27^. Fluorescent Nissl stains do exist, one good example being ethidium bromide^28^, but despite its low cost, this substance has a broad excitation and emission spectrum that would render the use of other stains more challenging^29^ as well as possessing toxic and carcinogenic properites^30,31^. More recently other fluorescent alternatives have been developed such as NeuroTrace®, a fluoro-Nissl based fluorescent marker that allows for detection of Nissl bodies comparable with standard immunofluorescent techniques^32^.

By understanding the similarities between Nissl staining and THT staining, namely their specificity for the same cellular structures, we decided to test if THT can achieve the same effects of NeuroTrace® for the fluorescent staining of neuronal cell bodies. Successful development of this method will afford a low-cost, easily applied and broadly available fluorescent alternative for the detection of neuronal cell bodies in fixed brain tissue for neuroscience research. We have also examined the use of THT in staining AD mice and judge whether it is suitable or not for amyloid beta detection. The findings of this research further expand the use of THT beyond just amyloid detection, and surprisingly identifies some previously undocumented photochemical properties of THT.

## Materials and methods

All experimental reagents used are listed in Table 1.

**Table 1:** Experimental reagents.

Reagent	Supplier	Product number
Microscopy		
Thioflavin T (THT)	(MedChemExpress)	HY-D0218
Anti Iba1, Rabbit 1° Antibody	FUJIFILM Wako	019-19741
Anti GFAP, Rat 1° Antibody	Thermofisher Scientific—Invitrogen™	130300 (2.2B10)
Alexa Fluor™ 647 2° Antibody	Thermofisher Scientific—Invitrogen™	A-31573
Alexa Fluor™ 594 2° Antibody	Thermofisher Scientific—Invitrogen™	A-21209
ProLong™ Glass Antifade Mountant	Thermofisher Scientific—Invitrogen™	P36980
NeuroTrace™ 640/660 Deep-Red	Thermofisher Scientific—Invitrogen™	N21483
Hoechst 33342	Thermofisher Scientific	62249
Microscope Slide: Superfrost™ Plus	Epredia™	J1830AMNZ
Coverslip: 24 × 60 mm #1 (0.13–0.16 mm)	Thermo Scientific—Menzel™	BB02400240A153FST0
Cell culture		
Fetal Bovine Serum—Paraguay	Sigma-Aldrich	F7524
DMEM/F-12, GlutaMAX™ Supplement	Gibco™	31331093
Penicillin–Streptomycin	Sigma-Aldrich	P4458
Sodium pyruvate solution	Sigma-Aldrich	S8636
L-Glutamine solution	Sigma-Aldrich	G7513
2-Mercaptoethanol	Gibco™	31350010

### Animals and tissue preparation

Female C57BL/6 mice (3–5 months old) or female APP NL-G-F Alzheimer’s disease mice^33^ (14 months old) were euthanized using isoflurane and subject to cardiac perfusion with ice cold PBS. Brains were extracted and directly placed into 5 ml of 4% paraformaldehyde fixative in PBS overnight, after which brains were subject to a sucrose gradient dehydration with a 20% w/v solution of sucrose in PBS for 24 h and then a 30% w/v solution for an additional 24 h. The brains were then removed, bisected along the sagittal plane and then placed in optimal cutting temperature compound (OCT) within a small square mold, then frozen over dry ice. These OCT frozen brains were cryo-sectioned at a thickness of 18 µm using a Leica CM 1850 cryostat and were then stored at -20°C until required for staining. APP NL-G-F samples were taken from mice which had received several intra-cisternal injections of PBS. All aspects of the study involving animals were conducted in accordance with ARRIVE guidelines and with the ethical permits approved by the Stockholm Animal Research Ethics board, Sweden.

### Staining procedures

THT (MedChemExpress) was dissolved in ultrapure Milli-Q® water at a concentration of 10 mM and passed through a 0.2 μm filter to form the stock solution and applied within 15–30 min of solution preparation. To form the working solution, the stock concentration was diluted to a concentration of 50 μM in PBS. Brain tissue cryosections samples were stained as follows: for protocol A for neuronal cell bodies and nucleoli, each sample being incubated for 20 min with 200 μl of staining solution then washed once for 5 min in PBS; for protocol B (Amyloid plaque) samples were washed twice for 5 min. Stained samples were then mounted using ProLong™ Glass antifade mountant (Thermofisher Scientific). All samples were mounted on Epredia Superfrost™ Plus Adhesion microscope slides using Menzel-Glazer 24 × 60 mm #1 glass coverslips. For samples that were co-stained with NeuroTrace™ 640/660 Deep-Red (NTDR) or Hoechst 33342 (both Thermofisher Scientific), NTDR being added at a 1:200 dilution and Hoechst 33342 at a 1:5000 dilution, both in PBS and incubated for 20 min.

For antibody staining, cryosectioned samples were first allowed to thaw for 15 min at RT then washed once in PBS for 10 min. This was followed by a 30 min incubation in blocking buffer (1% Triton-X100 with 1% bovine serum albumin (BSA)) followed by incubation with the primary antibody at 4 °C (IBA1 1:400, GFAP 1:200) overnight. The slide was then washed 2 × in PBS for 5 min and then incubated with the secondary antibody 1:400 in blocking buffer for 2 h in a dark humid container at room temperature. After this time, samples were washed once in PBS for 10 min and then subject to further staining before application of THT and Hoechst 33342. Images from slides were obtained using an LSM 880 confocal microscope (Carl Zeiss) and Zen Black software. Further information on excitation and emission spectra for different channels are presented in Supplementary S1A. Additional images were taken using the Echo Revolve R4 fluorescent microscope and detailed where relevant.

### Cell culture and live THT staining in vitro

BV2 microglia and C8D-1A astrocytes were cultured in DMEM F-12 culture media (Gibco) supplemented with 1% penicillin/streptomycin, sodium pyruvate (5 mM), l-glutamine (5 mM), 10% FBS (all Sigma-Aldrich) and 2-mercaptoethanol (0.2 mM) (Gibco) in T-75 flasks and used from passages 1–4 before restarting the culture. All BV2 microglia and C8D-1A astrocytes were plated at a density of 2 × 10^4^ cells per well in 96 well plates (Corning Costar) in 100 µl of complete media.

For live cell staining, culture media was replaced with 100 µl of 50 µM THT with 1:5000 Hoechst 33342 solution in DMEM/F-12 culture media without FCS for 15 min at 37 °C. This solution was pre-warmed in a water bath at 37 °C. Cells were directly imaged without removal of the media using a Biorad ZOE fluorescent cell imaging system in the green channel for THT (Excitation: 480/17, Emission: 517/23 nm) and blue channel for Hoechst 33342 (Excitation: 355/40 nm, Emission: 433/36 nm)^34^. All images were taken first using the green channel and then the blue channel to avoid the blue light photo enhancement effect.

### Confocal laser scanning microscopy, spectral imaging & fluorescent microscopy

**Fig. 1:**
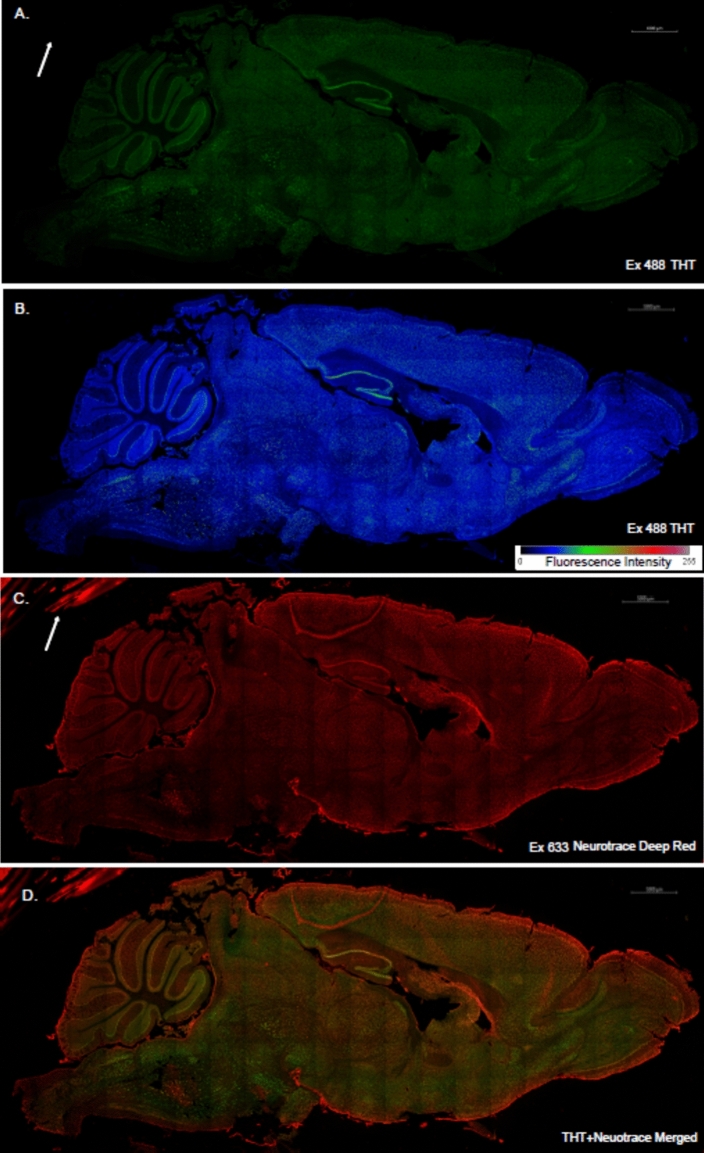
Representative staining of a mouse brain using THT and NTDR following protocol 1 at 10 × magnification. (**A**,**B**) THT intensity with 488 nm laser excitation in green pseudo color image (**A**) and heat map (**B**). (**C**,**D**) NTDR image with a 633 nm laser (**C**) and merge with THT (**D**). Scale bar: 1000 µm.

**Fig. 2:**
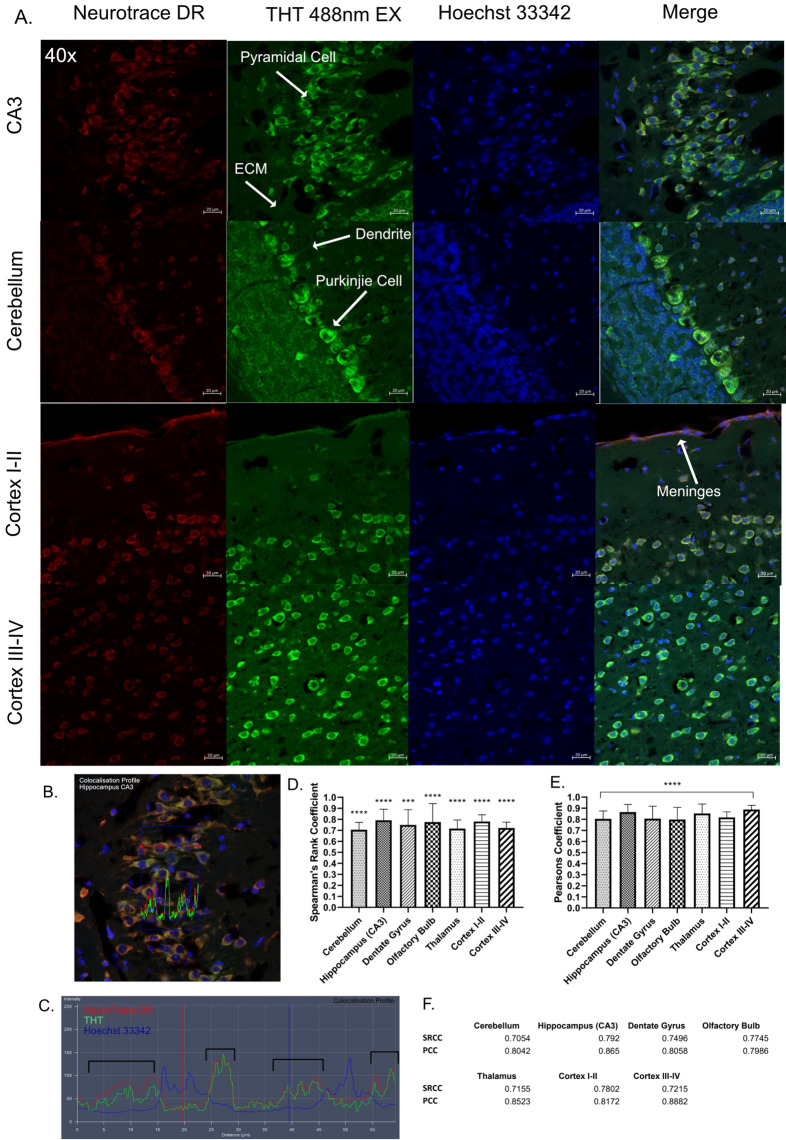
Co-localization analyses of brain regions stained with THT and NTDR, (**A**) Representative images of various brain regions in the brain section stained with NTDR, THT and Hoechst 33342. Scale bar: 20 µm. (**B**) Representative image of the CA3 region of the hippocampus for the intensity line profiles. (**C**) Comparison of line profiles between THT, NTDR and Hoechst 33342. Red: NeuroTrace DR (NTDR), Green: THT, Blue: Hoechst33342. Black brackets indicate the co-localization of NTDR and THT. (**D**) Pearson’s correlation analysis between THT and NTDR in various brain regions n = 5/6, (**E**) Spearman’s Rank Coefficient analysis for each of the brain regions (n = 6 all regions except Cerebellum & hippocampus n = 5, Average of all PCC and SRCC results respectively, an average score of > 0.7 was considered as evidence of co-localization for the PCC and SRCC, (**F**) Tabulated average SRCC and PCC corresponding to (**E**). A one sample T-test was conducted for each brain region in D,E.

**Fig. 3:**
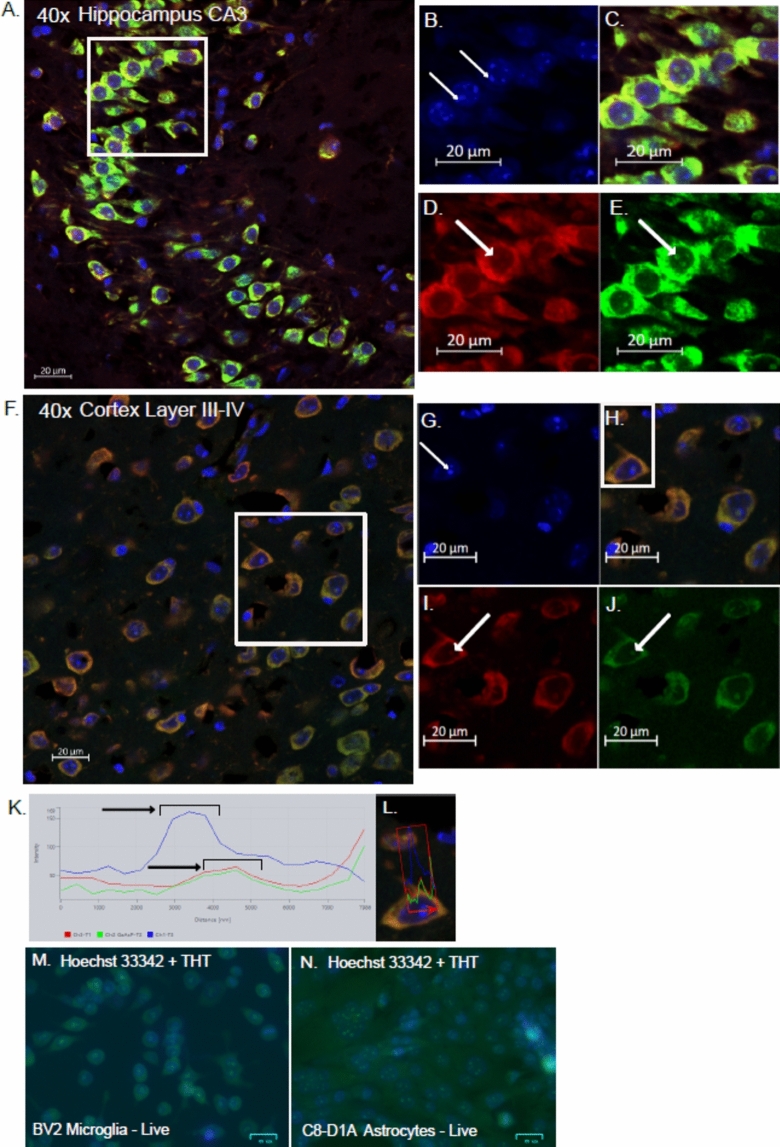
THT as a nucleolar stain in fixed tissue and live cells, (**A**) Representative images of the hippocampus. Blue: Hoechst33342, Red: NTDR, Green: THT. (**B**-**E**) Cropped image at white square area shown in (**A**). (**B**) Hoechst 33342 with arrows indicating chromatin structures. (**C**) Cropped merge image. (**D**) NTDR with arrow indicating faint nucleolus. (**E**) THT at 488 nm excitation with arrow indicating nucleolus. (**F**) Representative images of the cortex layers III-IV. Blue: Hoechst33342, Red: NTDR, Green: THT. (**G**–**J**) Cropped image at white square area shown in (**F**). (**G**) Hoechst 33342 with arrows indicating chromatin structures. (**H**) Cropped merge image. (**I**) NTDR with arrow indicating nucleolus. (**J**) THT at 488 nm excitation with arrow indicating nucleolus. (**K**,**L**) Representative spectral intensity profile of an example nucleus in the white region of (**H**), (**M**,**N**) Representative image of live BV2 microglia (**M**) and C8-D1A astrocytes (**N**) staining with Hoechst 33342 and THT.

**Fig. 4:**
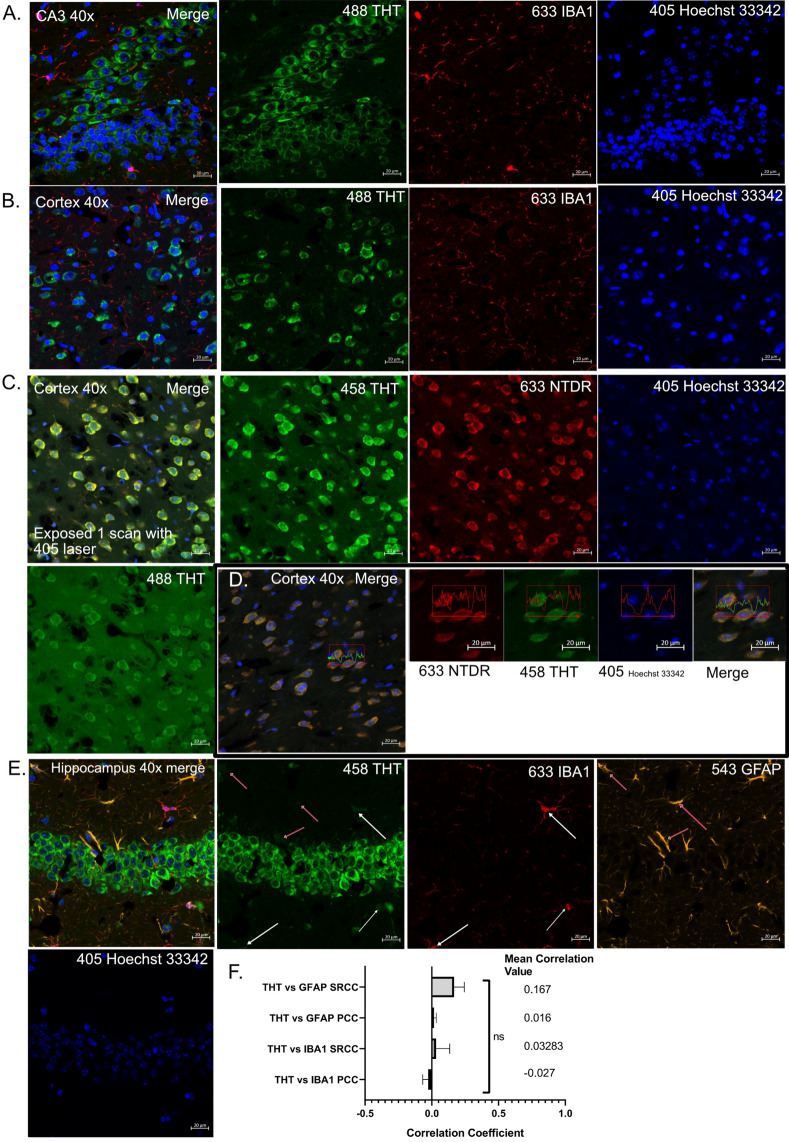
THT is compatible with antibody based fluorescent techniques. (**A**,**B**) Representative images of areas stained with THT, IBA1 (microglia/macrophage marker) and Hoechst 33342 at CA3 region (**A**) and Cortex (**B**). (**C**) Cortex imaged once with a 405 nm laser and then imaged again with THT, NTDR and Hoechst 33342 with THT excited by both 488 and 458 lasers. (**D**) Cortex stained with THT, NTDR and Hoechst 33342 demonstrating the spectral intensity overlap between THT excited with the 458 nm laser and NTDR, (**E**) Hippocampus stained with IBA1, GFAP, THT and Hoechst 33342, 40 × magnification, white arrows indicate IBA1^+^ microglia that partially stain positive for THT, red arrows indicate GFAP^+^ Astrocytes that microglia that partially stain positive for THT, (**F**) SRCC and PCC analyses for THT vs IBA1 or GFAP with corresponding mean values, pooled data from 2 experiments each with n = 3, Excitation laser wavelengths appear beside the stain used. A one sample T-Test was conducted for F.

**Fig. 5:**
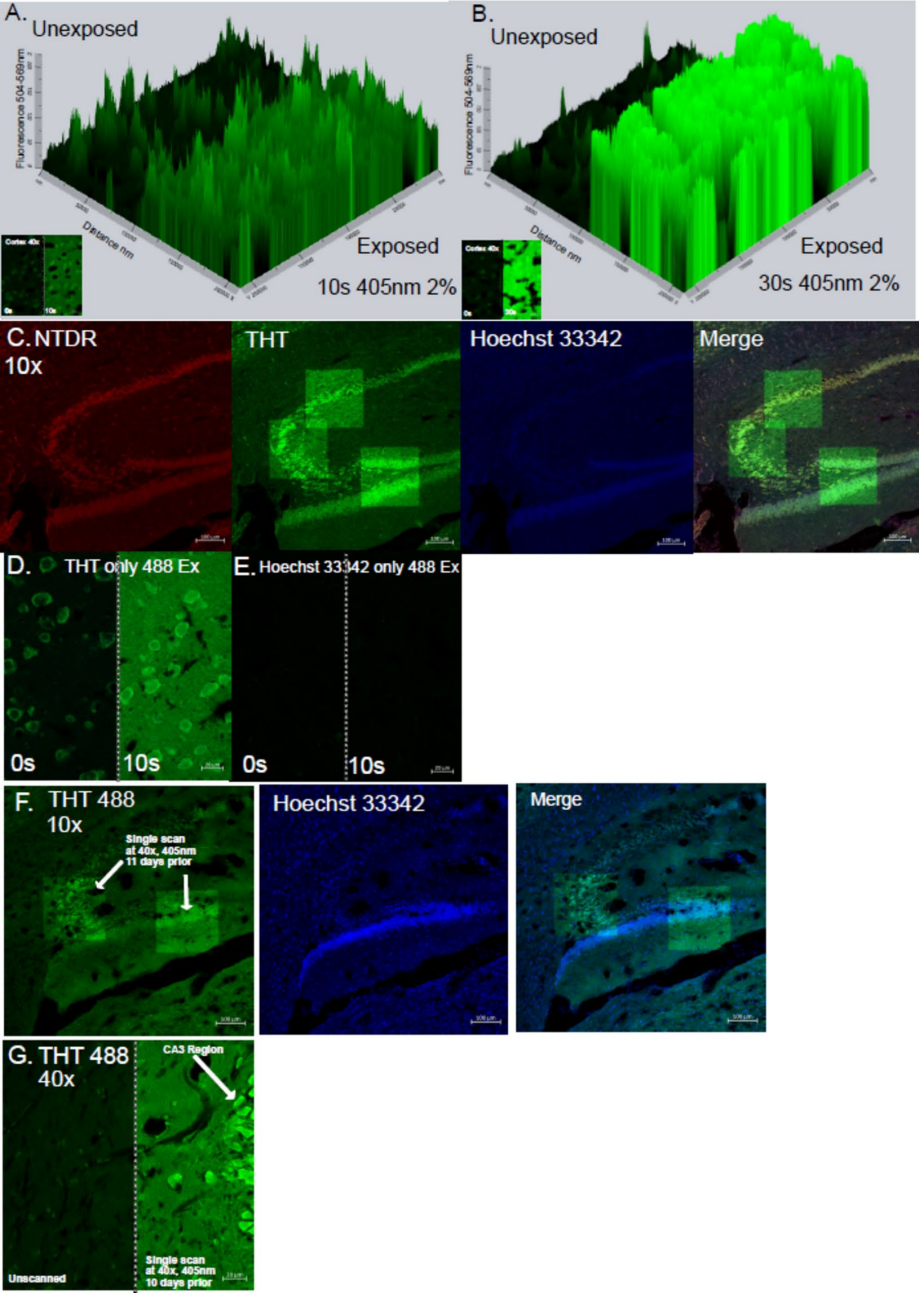
THT photo-enhancement by blue-light exposure. (**A**,**B**) Representative 2.5D images of THT-stained cortex imaged using the 488 nm laser after the exposure of right half side to a 405 nm laser scanning (770 µW) for 10 s (**A**) and 30 s, 40 × magnification (**B**). (**C**) Hippocampus stained with THT, NTDR and Hoechst 33,=342 (each 10 × magnification) imaged immediately after the 405 nm-laser-induced photo-enhancement at 3 regions on the hippocampus using 40 × magnification. (**D**,**E**) Sample stained only with THT (D) or Hoechst 33342 (**E**) imaged with the 488 nm laser without/with the exposure of the 405 nm laser for 10 s. (**F**) Hippocampus previously exposed to 405 nm laser by 40 × magnification 11 days prior to imaging with a 488 nm excitation laser and 10 × magnification. (**G**) Sample previously exposed to 405 nm laser by 40 × magnification 10 days prior to imaging with the 488 nm excitation laser and 40 × magnification.

Confocal Laser Scanning Microscopy (CLSM) imaging was performed using a LSM 880 confocal microscope (Carl Zeiss) and Zen Black software (Carl Zeiss)) equipped with laser lines (405 nm, 488 nm, 633 nm), objective lens (C-Apochromat, 40 × , 1.2 N.A., Corr and Plan-Apochromat 10 × /0.45 M27), a gallium arsenide phosphide (GaAsP) detector and photomultiplier tube (PMT) detectors. Hoechst 33342, and NTDR were excited using the 405 nm and 633 nm lasers, respectively. THT was excited using the 458 nm and 488 nm lasers depending on the experiment. The pinhole size was variable for each laser for Figs. 1, 2, 3, 4 and 5 which did not require direct comparison of emission spectra with 3.31–4.2 AU for Hoechst 33342, 2.81AU for 458 nm, 2.24 AU for 488 nm, 2.15–2.63 AU for 633 nm. The detection wavelength was 410–469 nm for Hoechst 33342, 504–549 nm for THT excited by the 458 nm laser, 504–569 nm for THT excited by the 488 nm laser, 647–759 nm for DNTR excited by the 633 nm laser, 638–724 nm for IBA1 (Alexa fluor 647) excited by the 633 nm laser, and 585–657 nm for GFAP (Alexa fluor 594) excited by the 543 nm laser respectively. To avoid overlapping signals between channels, multi-color imaging was performed in the multi-track mode.

Spectral imaging was also carried out using the same confocal microscope. Before spectral imaging, excitation laser power (458 nm, 488 nm, 514 nm) was calibrated to 5.00 µW (± 0.05 µW) on the 40 × water-immersion objective lens using a Newport optical 1917-R laser power meter to compare the fluorescence intensities obtained with different wavelengths of excitation lasers. The pinhole size for these experiments for each laser was kept at 91.5 AU, the 405 nm laser (770 µW) was scanned with 2.05 µs pixel dwell time on a random area of cortex for the blue light photo-enhancement effect. After the enhancement, the field of view was moved to the left so as to have the left half as the unexposed side and the right half as the exposed side. This field of view was then subject to spectral imaging with 3 nm resolution from a range of 451–721 nm for each of the lasers, and the areas exposed to the blue light were scanned only once.

Zen lite (3.4) software was used to analyze the spectral imaging data. A 10 µm circular region of interest (ROI) was placed on identifiable cells or surrounding extracellular matrix and then the average emission profile within ROIs were measured. This was done in duplicate for both cells and non-cells for both exposed and unexposed halves of each image, with a minimum of 3 repeats for each laser setting and with the experiment being repeated. The ROIs were selected at random using the 458 nm excitation generated emission profile due to the clear distinction between cells and surrounding tissue. The exact layout of the selected areas were then copied onto the lambda profiles of the 488 nm and 514 nm profiles for the respective area to better ensure comparability between results.

For tests on the effect of blue LED light on THT photo enhancement and photo bleaching, fluorescent imaging the Echo Revolve R4 microscope was employed. Each area of the brain was subject to 10% blue LED illumination power at 40 × magnification for 60s using a DAPI filter with Ex:380/30 and Em:450/50 (Dichroic mirror) Dm:425. The images were then collected using a FITC filter with Ex: 470/40 EM:525/50, Dm: 495.

### Correlation analyses

In order to determine correlation, FIJI software was used with the EzColocalization plugin^35^. Thresholds were determined using the automatic threshold algorithm with minor manual adjustments made if necessary, all thresholding being recorded for each image analyzed. After thresholding both the Pearson’s correlation coefficient (PCC) and Spearman’s rank correlation coefficient (SRCC) were determined using EzColocalization’s analyze function. SRCC and PCC were chosen due to their complementary nature and the arguable superiority of PCC over the Mander’s overlap coefficient (MOC) made by Adler & Parmyrd^36^. This process was repeated for the whole image of all areas of the brain analyzed and for all images analyzed. Average correlation sizes 0.7 and higher were considered positive for colocalization according to generalized standards for interpreting correlation size^37^, these were then tested for significance using a one-sample T test to derive the significance value corresponding to each average correlation size value.

Supporting methods were also employed for co-localization. Firstly, by using spectral profile mapping using Zen lite (3.4) software’s profile tool and drawing a line of measurement across cells or area to obtain the intensity profiles for each fluorophore measured. In this way, qualitative assessments were made by comparing the correlation of the different peaks for each fluorophore and by visual examination of the images. Secondly, spectrofluorograms were obtained using Zen Black software and correlation was also qualitatively assessed.

### Graphics and statistics

All graphs were made using GraphPad prism 8.0 software, a one sample T test being conducted for the PCC and SRCC analyses for the co-localization tests. Tests with 2 groups were conducted using an unpaired T test. P values of < 0.05* were considered significant and were designated using the GraphPad prism (GP) style^38^. Data is presented as mean ± SEM and all experiments have been conducted at least in duplicate. Representative images were analyzed in at least triplicate and Illustrations were made using Biorender software.

## Results

### THT stains neuronal cell bodies in fixed brain tissue

In previous experiments we had decided to apply 50 µM THT onto fixed tissues for staining of amyloid plaques by THT for 20 min. This provided a clear THT signal that was neither too bright nor dim compared with non-staining tissue (data not included). Many structures were strongly and selectively stained by THT, even in 4 month-old C57B/6 mouse brains where no amyloid pathology was expected. The staining patterns were visible in certain layers of the pyramidal cells at the hippocampus, cortex and also in the purkinjie layer of the cerebellum (Fig. 1A,B). This suggests that THT stains ER-rich neuronal cells, much in the same way that Nissl staining does.

To confirm the neuronal cell stained by THT, we co-stained THT with the fluorescent neuronal marker NeuroTrace® deep red (NTDR), revealing a general co-localization, however in the outer meninges, and residual blocking pen marks (white arrow indicated), NTDR but not THT fluoresced brightly (Fig. 1C,D). To determine co-localization, we then took magnified images of various brain regions that included the CA3 region of the hippocampus, cerebellum, thalamus, dentate gyrus and olfactory bulb (Fig. 2A). Like NTDR, THT seemed to preferentially stain cells with large soma, such as pyramidal cells evident in the hippocampus and cortex as well as cerebellar purkinjie cells^39,40^. THT also poorly stained the surrounding extracellular matrix thus provides a good visible contrast between them and the cells. However, THT stained dendrites but not axons (Fig. 2A). A line profile of a representative image at the CA3 region of the hippocampus demonstrates good correlation between NTDR and THT, but not Hoechst 33342, indicating colocalization of NTDR and THT (Fig. 2B,C). The PCC and SRCC results further indicate the co-localization of NTDR and THT across the various brain regions (Fig. 2D,E), which may suggest that THT could be used as a direct alternative to NTDR. One difference we noticed is that NTDR seems to stain the meningeal layer stronger than does THT, although this has only been visually assessed in a qualitative manner (Fig. 2A Cortex layers I-II white arrows).

### THT staining reveals nucleoli within both fixed tissue and live cells

Aside from the staining of the cell bodies, we observed dotted structures at the nucleus in both NTDR and THT staining. Images of the CA3 region of the hippocampus (Fig. 3A–E) and at the cortex layer III-IV (Fig. 3F–J) clearly show smaller structures within the nucleus of these cells. Line profiles show that the peak for Hoechst 33342 does not coincide with the peaks for NTDR and THT which do coincide, outlining the difference of intranuclear staining these fluorophores provide (Fig. 3K,L). NTDR/THT-positive structures are distinct from the smaller and more numerous granules stained with Hoechst 33342 positive granules, which are likely heterochromatin structures^41^. This may suggest nucleoli staining by NTDR and THT due to the highly congruent staining pattern, clear visibility of the structures and that neuro-trace family of dyes are fluorescent Nissl based, for which Nissl is known to intensely stain the nucleolus.

We also tested THT staining of nucleoli in live cells to assess. Both cell types, BV2 microglia and C8-D1A astrocytes which are examples of cells in central nervous system (CNS) stained positively with THT in the cell bodies and the nucleoli. BV2 microglia are relatively small and had around 1–2 clearly visible nucleoli, whereas the C8-D1A astrocytes were larger and also had a higher number of 4–5 nucleoli per cell (Fig. 3M,N). The nucleolar staining of THT in both live cells and fixed tissue demonstrates its potential use in the study of nucleolar dynamics, which is highly relevant to proteostasis, although further optimization is needed for its use on live cells for this purpose^42^.

### THT staining is compatible with standard Immunofluorescent techniques and can be imaged with either 488 or 458 nm excitation lasers

The utility of THT staining also depends on its compatibility with standard antibody-based immunohistochemical methods, since it is extremely prevalent in modern histology. To determine that THT staining for neuronal bodies is compatible with such methods, we co-stained brain sections with THT and IBA1 as a microglia marker. Representative images of the CA3 region at the hippocampus and cortex indicate no colocalization between IBA1 and THT in both sections (Fig. 4A,B). Conversely, neuronal cell marker, NTDR clearly co-localized with THT (Fig. 2), clearly suggesting that both markers, THT and IBA1 separately stain neuronal and microglia cells respectively. Using the 458 nm laser was more suitable for THT excitation, the same co-localization being observed in cells with higher fluorescence signals (Fig. 4C,D). The outside of cells was also observable with 458 nm laser excitation, probably suggesting that THT binds to an extracellular matrix (ECM). In order to further identify possible glial cell staining by THT, we stained samples with THT, IBA1 and GFAP (astrocytes) to see if THT colocalized with these markers. We could observe that although there was no overall colocalization, it is possible to make out instances of faint THT staining that appear to mark the cell bodies of some microglia and astrocytes Fig. 4E,F.

### THT fluorescence intensity at 488 nm excitation displays long-lasting photo enhancement by pre-exposure to blue light

Unexpectedly, we acquired low contrast images using a 488 nm laser after 405 nm illumination for the Hoechst 33342 excitation, but this was unobservable using the 458 nm laser (Fig. 4C). To provide better understanding of the photo-enhancement effect of THT by blue laser illumination, we stained brain sections following protocol 1 and purposely exposed them to a blue laser. Compared to neighboring non-exposure regions, the 405 nm laser illumination yielded strong enhancement of THT fluorescence signals, and longer exposure enhanced even more with an exposure-dependent manner (Fig. 5A,B). Due to this enhancement, the previously clear neuronal bodies became increasingly hard to distinguish from the surrounding tissue (inserted images in Fig. 5A,B). Neither NTDR nor Hoechst 33342 experienced such changes upon exposure to blue light, indicating that they are very different compounds with different photochemical properties (Fig. 5C). The enhancement was unobservable in samples stained with Hoechst 33342 alone, but staining with THT alone had such effects, suggesting a THT-specific effect (Fig. 5C–E). This was a long-lasting effect as it was still evident after 10 days (Fig. 5F,G).

### Spectral profiling characterizes the blue light photo enhancement effect of THT in stained tissue

**Fig. 6:**
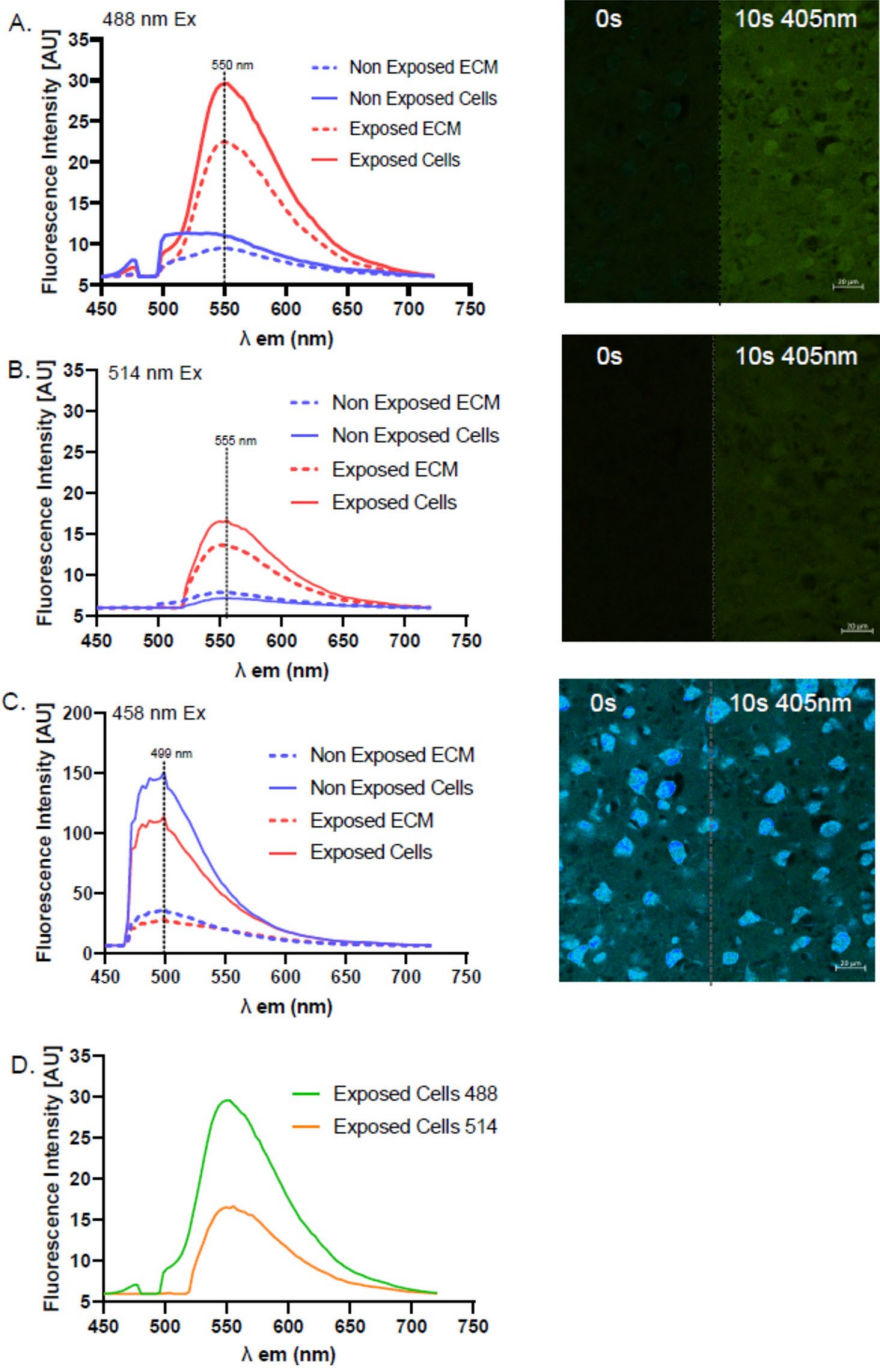
Spectral imaging analysis on blue-light photo-enhancement. (**A**–**D**) Fluorescence spectral profile of cells and surrounding tissue with or without pre-exposure to 405 nm laser (770 µW) 10 s. Spectral imaging was performed using the 488 nm (**A**), 514 nm (**B**) and (**C**) 458 nm laser excitations. (**D**) Spectral profile comparison between the exposed cells at 488 vs 514 nm excitations. Green: 488 nm excitation laser, Orange: 514 nm excitation laser. Representative lambda profile overlays on the right, vertical dashed bar represents the peak fluorescence intensity from exposed cells (rounded to 3 significant figures). Each experiment with 3 repeats per wavelength, each graph made by pooling 3 experiments.

To characterize the emission spectral properties of this photo-enhancement effect at random areas of cortex tissues stained with THT, spectral imaging was performed with 3 excitation wavelength lasers: 458 nm, 488 nm, and 514 nm after the laser power calibration to same laser power illumination to ensure comparable results (see “Methods” and Supplementary S1B). The results recapitulated that both surrounding tissue and the neuronal bodies all had increased fluorescence intensity after the photo-enhancement induced by the blue laser. Both the 488 and 514 nm lasers gave an increase in the fluorescence peak maximum at approximately 550–555 nm, which was several fold higher than in unexposed areas (Fig. 6A,B). Interestingly, the blue laser-induced photo-enhancement was not observed with the 458 nm laser, whereby the fluorescence peak was located at 499 nm and the neuronal bodies were clearly visible and distinguishable from surrounding tissue. However, there was a small amount of photo-bleaching evident in the 458 nm condition (Fig. 6C). The 488 nm laser seems to give the strongest signal, indicating the peak excitation of this blue laser-induced effect being closer to 488 nm rather than at 514 nm excitation, which indicates the peak excitation lies somewhere around 488 and 514 nm excitation and less towards 458 nm (Fig. 6D). It is important to note that the 458 nm excitation provided a much higher emission signal, around fivefold higher than with the 488 nm laser and tenfold higher than the 514 nm laser due to suitable laser wavelength of THT (Fig. 6A–C). The full spectral imaging sets for each wavelength is presented in the Supplementary S1C,D.

### Blue light exposure simultaneously photo-enhances THT stained tissue at 488 nm excitation and photo-bleaches at 458 nm

**Fig. 7:**
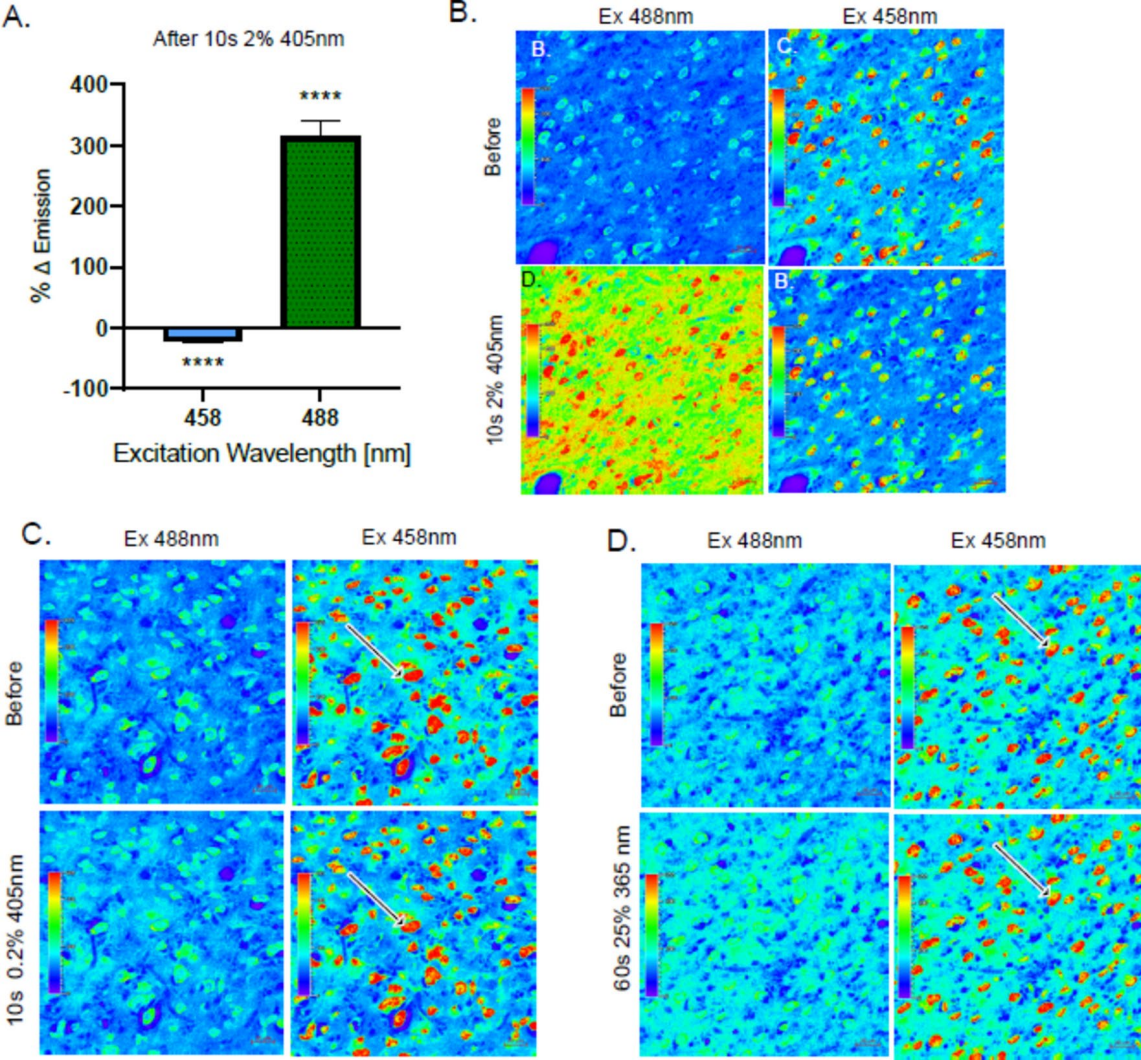
THT blue light photo enhancement and photo bleaching. (**A**) Change in emission intensity of cortex sample calculated from the images shown in (**B**), n = 3 pooled from 2 experiments. (**B**) Representative heat maps acquired by 458 nm/488 nm excitation laser before and after the 405 nm laser illumination (2%, 770 µW) for 10s by 40 × magnification lens. (**C**) Representative heat maps acquired by 458 nm/488 nm excitation laser before and after the 405 nm laser illumination (0.2%, 77 µW) for 10 s by 40 × magnification. (**D**) Representative heat maps acquired by 458 nm/488 nm excitation laser before and after the 365 nm halogen lamp (25%) for 60 s by 49wf blue filter (Carl Zeiss) and 40 × magnification. Arrows represent examples where loss of signal is evident after blue light exposure in the using 458 nm. A 2 sample T-test was conducted for A.

Since we had noticed a slight decrease in the fluorescence intensity after blue laser pre-exposure at 458 nm excitation after the spectral imaging, we decided to follow up on this observation (Fig. 6C). Areas of the cortex were exposed to blue laser again to recapitulate the effect of photo-enhancement and potential photo-bleaching (Fig. 7A,B). The expected photo-enhancement effect at 488 nm was evident, but also a lesser yet still significant photo-bleaching effect at the 458 nm wavelength. This effect was reduced by reducing the 405 nm laser power from 2% to 0.2%, which led to a reduced yet still visible bleaching (Fig. 7A–C). Furthermore, to test the effect of simply viewing the sample under blue light we exposed the sample to 365 nm blue light from the halogen lamp using Filter Set 49 (Carl Zeiss). We recorded a very slight photo-enhancement effect when later imaged with the 488 nm laser, and a very slight photo-bleaching at 458 nm when exposed for a longer duration (Fig. 7D). This is probably due to the lower excitation light density from the halogen lamp in comparison to the highly focused laser beam. It seems that the blue laser photo enhancement effect also extends to wavelengths in the UV spectrum (Fig. 7D). This effect was also confirmed using an a blue LED on a widefield microscope that confirms the effect is not limited to the imaging modalities on the confocal microscope (Fig S2).

### Protocol establishment: Aβ plaque or neuronal cell body/nucleolar stain

**Fig. 8:**
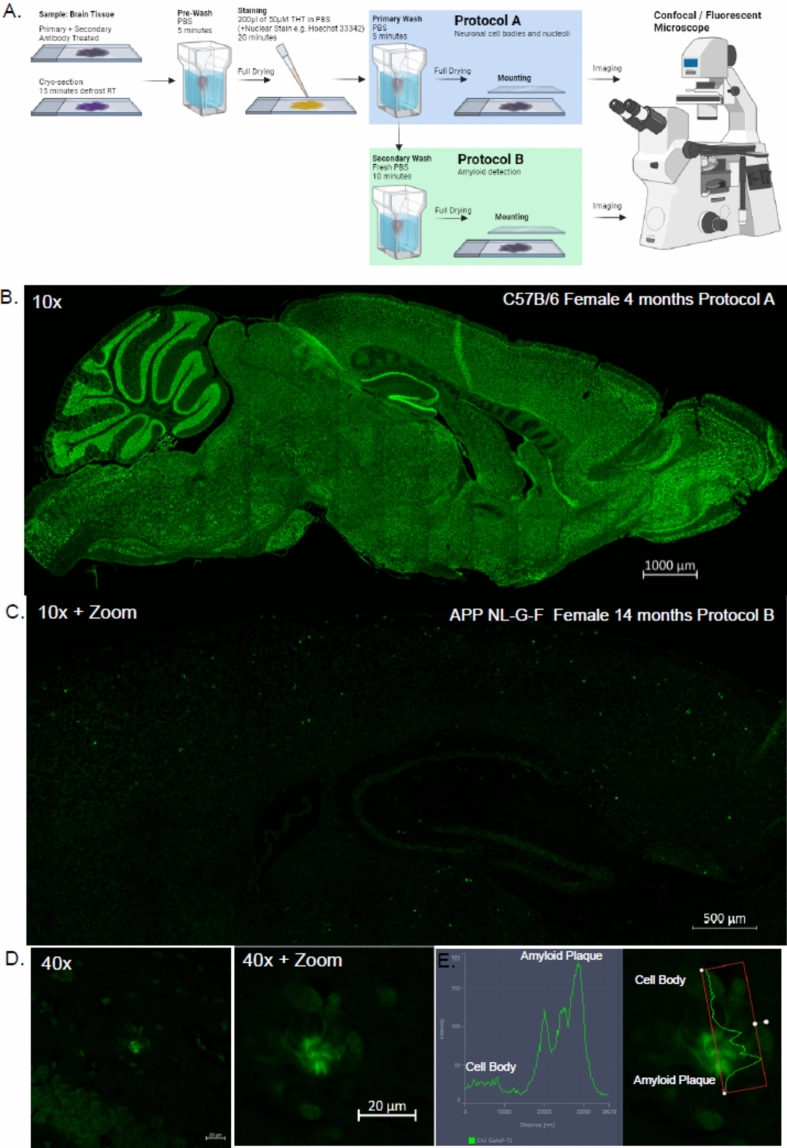
Establishment of THT staining protocols, (**A**) Illustration of THT staining protocols A and B with accompanying notes. (**B**) Representative image of a female 4 month-old C57B/6 mouse brain section viewed on the sagittal plane stained using protocol A imaged using the 458 nm excitation laser and 10 × magnification. (**C**) Representative image of a female 14 month-old APP NL-GLF brain section viewed on the sagittal plane stained using protocol B imaged using the 458 nm excitation laser and 10 × magnification lens with digital zoom to highlight the cortex and hippocampus. (**D**) Representative image of a THT-stained plaque in the molecular layer surround the dentate gyrus taken at 40 × magnification (left) digitally zoomed in to demonstrate the fibrillary plaque structure (right), (**E**) Fluorescence intensity plot of THT stained plaque in (**D**) compared to a nearby stained cell.

We next tested THT staining of AD mouse CNS tissues from APP NL-G-F mice. Originally we attempted to follow a protocol outlined for THT Plaques used to stain brain sections of AD model GF-APPPS1 mice using a 1% solution (31.2 mM) in 50% ethanol solution, but could not find specific information regarding the “standard protocol” listed in their methods^43^. Upon attempts with a 1% THT solution for 20 min with subsequent washing steps, we found that this concentration yielded an extremely bright sample fluorescence for our cryosectioned samples, which made distinguishing structures difficult (data not included). We therefore kept modified our established protocol A. We added an additional wash step for protocol B as we had noticed that the fluorescence signal of THT can be attenuated by increasing washing steps, and we believed that THT would have a stronger binding affinity to these amyloid pathologies plaques based on older protocols and could thus be selectively stained^7,44^. Since amyloid plaques should be visible at 4 months in APP NL-G-F mice and increase with age, we chose 14 month- old mice to ensure good examples of plaque development^45^. We therefore modified protocol A by adding an additional 10 min washing step with PBS called protocol B (Fig. 8A). This additional washing step removes much of non-specifically bound THT from the tissue, allowing visualization of plaques with better contrast against neuronal bodies using the range indicator function in the confocal microscope. A clear image of the brain and the neuronal cell bodies could be obtained using protocol A with the 458 or 488 nm lasers (FigS. 1A,B, 8B). Furthermore, with protocol B the distribution of the THT stained plaques were clearly visible and were distinct from the less intensely stained neuronal cell bodies (Fig. 8C–E). A closer inspection of these plaques revealed a fibrillar structure that fluoresced at an intensity several magnitudes higher than in local cell bodies (Fig. 8D,E). Protocol A should be followed to use THT as a neuronal/nucleolar, while for staining of amyloid plaques additional washing steps and use of protocol B is recommended. Washing steps may be further increased or decreased depending on the users samples, and we recommend using some non-essential samples to test this. We must also state the caveat that we have only applied these to samples that have undergone fixation, sucrose dehydration and OCT embedding before sectioning with a cryostat. Paraffin embedded samples may require a different approach. The full list of materials used is mentioned in the methods section in Table 1.

## Discussion

In this study we demonstrate the application of THT as a robust marker for neuronal cell bodies that is equivalent in this regard to fluoro-Nissl based markers such as NeuroTrace®deep red (NTDR). It only faintly stains some surrounding glial cell bodies and can be used as a stain to map brain tissue samples. When using THT in this capacity, based on our findings we advise to first view the sample using a 458 nm laser for orientation and structural identification with THT rather than at 405 nm for Hoechst 33342 or any ultraviolet filter sets, and to use a lower blue laser power with reduced exposure time when it comes to capturing images to prevent the photo-enhancement or photo-bleaching effects. Furthermore, when setting up any acquisition that requires multiple channels we recommend to save the blue channel for last.

Furthermore, we demonstrate that THT also stains nucleoli in both fixed tissue and live cells, which is potentially useful in the monitoring of nucleolar dynamics that is a core feature of many pathologies. Further tests could be done to validate its use in testing nucleolar dynamics in conditions of inflammation, pathogen exposure and general cell stress, and also as a tool to examine the nucleolus in fixed tissue, especially with the use of super resolution microscopy.

For unknown reasons THT seems to be used less often for amyloid detection in histological samples in favor of other dyes such as Thioflavin-S (THS), Congo-red and its more modern derivative methoxy-X04, or antibody-based methods^46,47^. We speculate that this might be due to the historical bias or perhaps the unwanted effect of neuronal cell body and nucleoli staining we have outlined, but we demonstrate the utility of THT in light of this complication.

Another question we have attempted to understand is which cells THT binds to and why. Judging by its almost exact correlation to Nissl staining, it likely stains pyramidal neurons due to their richness in Nissl bodies. As previously mentioned, these Nissl bodies comprise of rough endoplasmic reticulum enriched with ribosomes and as such they are the primary protein synthetic machinery of the cell^48,49^. These pyramidal neurons receive many synaptic inputs and transmit them along their axons for long distances, they are the most numerous excitatory cell and they thus have large amounts of ER and related machinery in order to fulfil high protein synthesis and signaling demands^50,51^. Since the ER is a site that is central to protein synthesis, folding and quality control, misfolded proteins regularly accumulate there, and this could be one reason why THT binds Nissl bodies and also the nucleoli, that are crucial for protein quality control^22,52^. THT also increases in fluorescence in the ER of cells undergoing stress, indicating it could be binding to increased misfolded proteins or else there is some intrinsic structure both within the ER and nucleoli which binds THT. However, it may also be that THT itself in high enough concentrations can accelerate protein aggregation^3,17^. Another possibility is the ability of THT to increase in fluorescence intensity upon binding RNA, and as both the ER and nucleoli are RNA-rich structures, we believe this to be more likely^53,54^. A combination of these different factors may also be possible.

We have also revealed some interesting physical properties of THT that upon exposure to blue light an interesting photo-enhancement of the sample occurs that is evident when stimulated with lasers at 488 nm and 514 nm, but not at 458 nm. There does not seem to be any indication of this phenomenon in the literature although there is one study that describes blue light as a trigger to facilitate changes in membrane potential of mitochondria^20^. We recommend for neuronal staining purposes to use the 458 nm laser (as it is barely affected by this blue light, save for photo-bleaching) and not the 488 nm laser, since although the 488 nm laser can detect the neuronal bodies the blue light photo-enhancement effect will make it more difficult to distinguish between true signal and background.

We have not defined the exact cause of this photo-enhancement effect, although we believe that it may be related to THT excimers that are in the solution. An excimer is an excited dimer of a molecule that expresses different fluorescent properties. THT and structurally related compounds such as 2-phenylindone are capable of forming excimers characterized by a red-shift in the emission spectra^55^. One study reported that by increasing the THT dye concentration, a concentration-dependent red shift (490 to 570 nm) was evident in 3–300 µM THT concentrations using 412 nm light excitation^56^. Although we used a fixed concentration of 50 μM that lies in between this range, we obtained a similar trend in results without changing concentrations. Through pre-exposure to a blue laser we could obtain an emission peak of 495 nm after 458 nm laser excitation and a 550 nm emission peak after 488 nm or 514 nm laser excitation. If related to the previous findings for THT excimers, it is possible that some aspect of blue laser exposure facilitates the formation of these THT excimers from monomers, thereby affecting the total fractionation of the solution and the resultant red shift of the average absorption and emission spectra.

Since the fluorescence increases are indiscriminate and not located to the neuronal cell bodies, it could be the result of these excimers that are non-specifically binding or resting above the substrate, which agrees with the previously mentioned study that it is not the THT excimer, but instead the THT monomer that is responsible for selective binding fluorescence to beta sheets^56^. Perhaps the blue light induces the THT monomers to dimerize and thus form excimers that fluoresce without binding to beta sheets, thus increasing the fluorescence signal and providing the characteristic red-shift, which would be more likely at the high concentrations such as 50 μM THT that we have employed herein. THT is also known to form micelles at above 5 μM, which themselves appear to increase fluorescence too and which may play a role^3^. The fact that with additional wash steps the fluorescence signal for THT could be extinguished from the neurons but not from the amyloid plaques suggests that the binding to the Nissl bodies is weaker than for beta sheets present in the amyloid plaques.

This property of THT means that care must be taken for use in conjunction with dyes that require excitation with 488 or 514 nm lasers, due to the blue-light-induced photo-enhancement effect. Since protocols regularly employ either DAPI or Hoechst33342 for nucleus identification, blue light exposure is highly likely to occur and so this effect will be evident^57^. We suggest the use of a far-red dye such as NeuroTrace® deep red or with IBA1 bound to an Alexa 647 secondary antibody, which both work well. We also recommend observing THT fluorescence by excitation with a 458 nm laser and not the 488 nm laser, although it is still possible to obtain images this way, and scanning with the 405 nm laser last if a multi-color image is required. Thus a 3- or 4-fluorophore setup can be obtained that is suitable for most staining purposes, making sure to omit the excitation from the 488 nm and 514 nm lasers and the general emission spectra from these lasers. One must also be careful not to view areas for too long when using blue laser or light, as there is noticeable photo-bleaching effect which could become an issue to consider if quantitative fluorescence data is required. This enhancement is also apparent using a 0.2% power 405 nm laser (typically used for imaging DAPI or Hoechst 33342) (Fig. 7C) which we have measured to be 78 µW. It may be possible that rather than the blue wavelength specifically inducing these effects, the high energy density of blue light induces these changes and that excitation of THT with any wavelength of light with sufficient power would induce similar effects. Experiments using directly comparable wattages of different lasers to the 405 nm laser are also warranted to further understand this effect.

The blue-light-induced photo-enhancement effect on THT is not necessarily detrimental. It could be used as a marker to detect UV or blue light exposure whereby a photo enhancement effect can be seen that can be applied to fields that study UV radiation, or else be incorporated into materials that require evidence of accumulated UV or blue light exposure for clinical or industrial application. Furthermore, this effect could be used for bio-cryptography, for instance via micro-printing using the focused blue-laser scanning^58^. Information could be stored in detail depending on laser width and erased by a blanket exposure of the entire sample. However, such suggestions require further investigation and development for any other practical use, but this study illustrates that THT exhibits physical properties that extend its use beyond a just protein aggregate and neuronal marker.

In conclusion, we have demonstrated a quick, simple and cost-effective method for the use of THT as a neuronal and nucleolar stain in fixed tissue employing the single wash protocol A. THT can be used to clearly distinguish between neuronal cell bodies and nucleoli against the background in a comparable way to existing but more costly fluoro-Nissl stains such as NeuroTrace® in a method that is also compatible with immunofluorescent staining. Furthermore, it seems that THT does not bind strongly to meningeal cells on border regions unlike NeuroTrace® (Figs. 1C,D, 2A), which will be an advantage if specific staining of those cells is also required and to limit non-specific binding in general. The staining protocol B works well for amyloid plaques by simply increasing washing steps in A that will preferentially remove the THT from the nucleoli and cell bodies and instead leave it bound to these plaques.

Aside from THT’s use in fixed tissue we also demonstrate its use in vitro with live cells, which opens the possibility of a novel method for studying of nucleolar dynamics in cell stress and inflammation settings^59,60^. Should this method be developed further, it could be a good alternative to more expensive and less selective existing RNA stains such as STYORNASelect or reliance upon cells that express fluorescent nucleolar reporters^59,61^. Furthermore, we have demonstrated the blue-light-induced photo-enhancement effect of THT that is possibly caused by increased THT excimer formation and outlined methods to avoid the interference of this effect with regards to the use of THT as a cell stain. This finding is also very interesting as it opens up the potential for this effect to be exploited in areas that require a photosensitive dye where UV or blue light may be encountered.

## Supplementary Information

Supplementary Figure S1.

Supplementary Figure S2.

## Data Availability

Raw data used to generate the figures are available from the corresponding author, Jin-Hong Min, upon request.
