# Publisher Correction: Thioflavin-T: application as a neuronal body and nucleolar stain and the blue light photo enhancement effect

**DOI:** 10.1038/s41598-024-82113-3

**Published:** 2024-12-20

**Authors:** Jin-Hong Min, Heela Sarlus, Sho Oasa, Robert A. Harris

**Affiliations:** Department of Clinical Neuroscience, Karolinska Institutet, Center for Molecular Medicine, Karolinska University Hospital, 171 76 Stockholm, Sweden

Correction to: *Scientific Reports* 10.1038/s41598-024-74359-8, published online 22 October 2024

In the original article, the typesetter omitted the below Reference, which is listed below as Reference 1.

1. Saito, T. *et al*. Single App knock-in mouse models of Alzheimer’s disease. *Nat. Neurosci*. **17**, 661–663 (2014).

As a result, in the Animals and tissue preparation section,

“Female C57BL/6 mice (3–5 months old) or female APP NL-G-F Alzheimer’s disease mice (14 months old) were euthanized using isoflurane and subject to cardiac perfusion with ice cold PBS.”

now reads:

“Female C57BL/6 mice (3–5 months old) or female APP NL-G-F Alzheimer’s disease mice^1^ (14 months old) were euthanized using isoflurane and subject to cardiac perfusion with ice cold PBS.”

As a result of the changes, the References have been renumbered.

The original Article has been corrected.

