# Supplementary material for: Thioflavin-T: application as a neuronal body and nucleolar stain and the blue light photo enhancement effect
Source: Sci Rep. 2024 Oct 22;14:24846. doi: 10.1038/s41598-024-74359-8 (PMC11496653; doi:10.1038/s41598-024-74359-8)
Supplement: Supplementary file 1 — Supplementary Figure S1. [file 41598_2024_74359_MOESM1_ESM.pdf]

S1.

A.

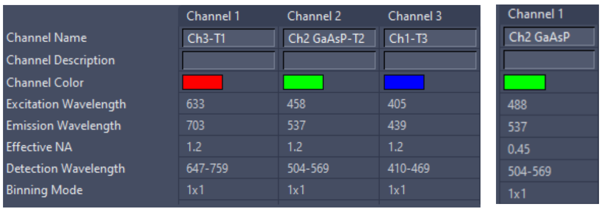

B.

Laser λ (nm)	Laser Power (%)	Wattage (μW)
458	6	5
488	0.84	5
514	1.95	5

C.

λ ex 458

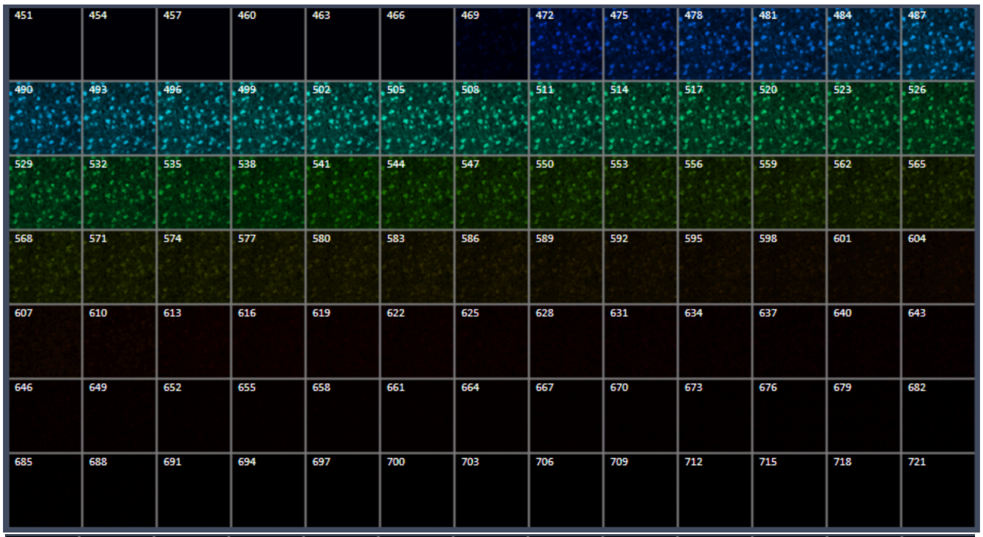

D.

λ ex 488

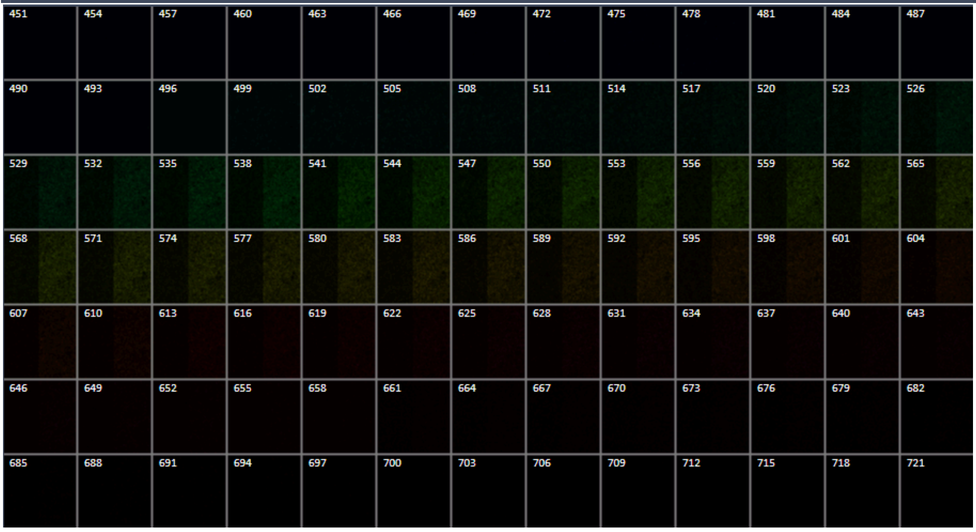

E.

λ ex 514

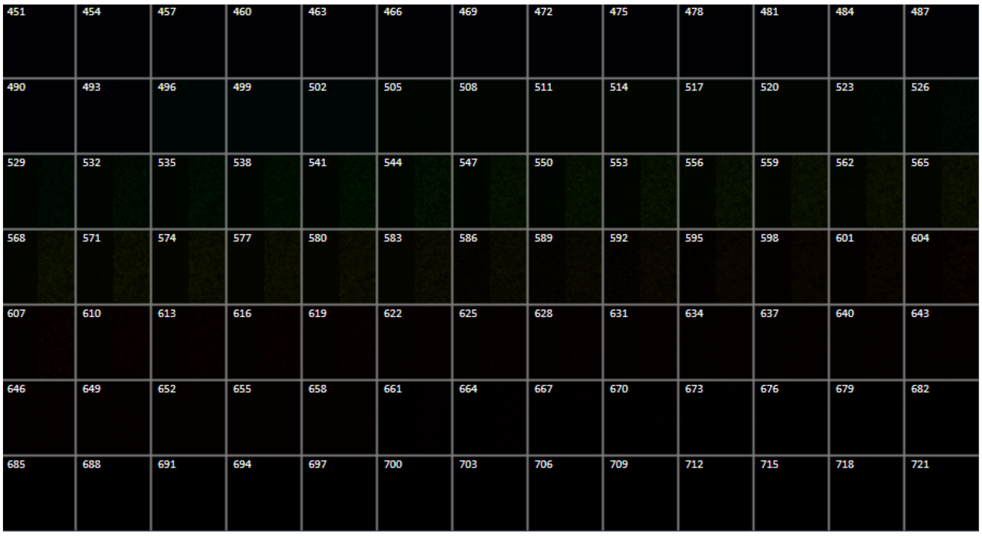

Figure S1. Imaging Details, (a) settings for excitation and emission spectra used for capturing images throughout the study, (b) calibrated laser powers for lambda imaging, (c-d) 91 image, 3nm interval Lambda imaging series for 458nm, 488nm and 514nm excitation lasers from a range between 451-721nm
