# Supplementary material for: Thioflavin-T: application as a neuronal body and nucleolar stain and the blue light photo enhancement effect
Source: Sci Rep. 2024 Oct 22;14:24846. doi: 10.1038/s41598-024-74359-8 (PMC11496653; doi:10.1038/s41598-024-74359-8)
Supplement: Supplementary file 2 — Supplementary Figure S2. [file 41598_2024_74359_MOESM2_ESM.pdf]

S2.

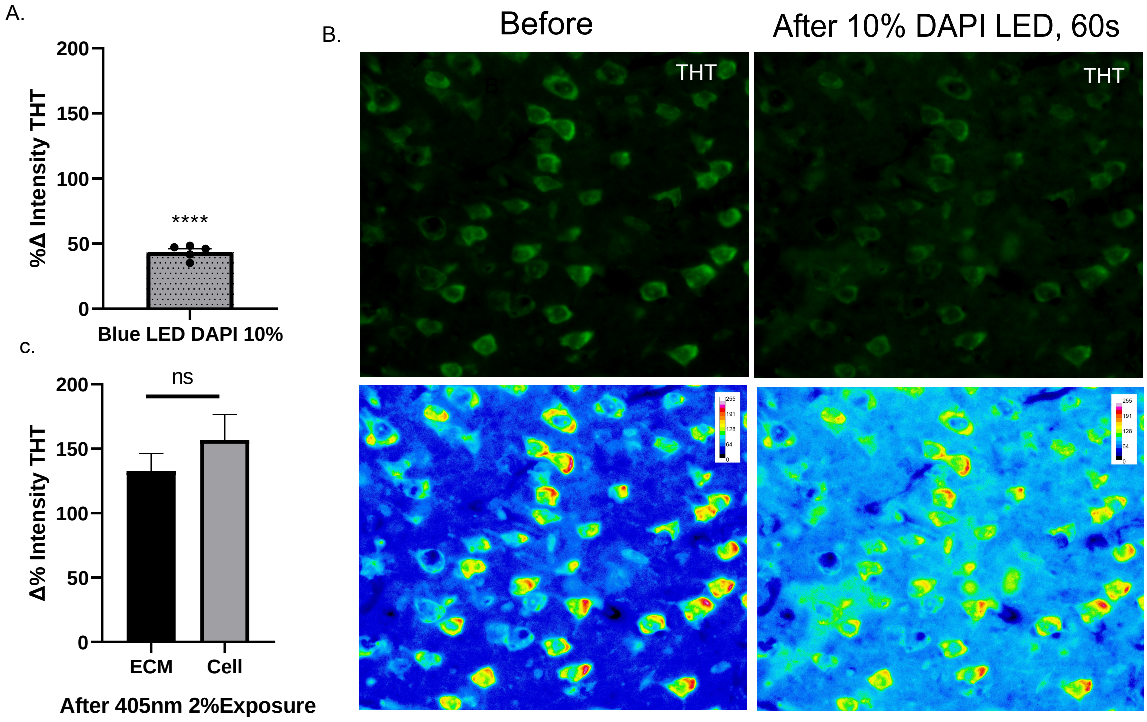

Figure S2. Supplementary information of the blue light photo enhancement/photo bleaching effect of blue light exposure on THT stained mouse brain tissue*. (A) n=5 Changes in fluorescence intensity of THT stained tissue samples exposed to a blue LED (10% power) for 60 seconds on a fluorescent microscope (Echo Revolve R4) 40x magnification, (B) representative images of (A), (C) changes in fluorescence intensity of 10 seconds 2% (770 µW) blue laser exposed THT stained extracellular matrix and THT stained cells compared to their respective non-blue laser exposed counterparts, excited by a 488nm laser and measured at the 555nm excitation peak 40x magnification, (data reanalyzed from the spectral imaging in figure 6A, n=3, pooled from 3 experiments).*
